# Supplementary material for: Emotional Distress in Portuguese Cancer Patients: The Use of the Emotion Thermometers (ET) Screening Tool
Source: Healthcare (Basel). 2023 Oct 6;11(19):2689. doi: 10.3390/healthcare11192689 (PMC10572115; doi:10.3390/healthcare11192689)
Supplement: Supplementary file 1 [file healthcare-11-02689-s001.zip › healthcare-2508224-supplementary.pdf]

healthcare

MDPI

Supplementary Materials

Table S1. Unadjusted generalized linear model results for the univariate relationship between patient characteristics, problem list, and distress.

	Distress	
	**Coefficient**	**Std. Err.**
**Demographic**		
Age [mean (SD)]	0.00	0.00
Female	0.12***	0.03
**Disease phase**		
Treatment	Ref.	
Survival	-0.01	0.03
Palliative	0.07	0.04
Diagnosis	0.05	0.04
Relapse	0.12***	0.05
**Practical**		
Housing	0.11***	0.03
Security	0.13***	0.03
Work/school	0.09***	0.02
Transportation	0.07	0.04
Childcare	0.06	0.05
**Family**		
Dealing with partner	0.08***	0.02
Dealing with children	0.10***	0.02
**Emotional**		
Worry	0.26***	0.07
Ferars	0.18***	0.03
Sadness	0.37***	0.04
Depression	0.27***	0.02
Nervousness	0.18***	0.03
**Spiritual**		
Relating to God	0.10***	0.03
Loss of Faith	0.15***	0.03
**Physical**		
Pain	0.12***	0.02
Nausea	0.09***	0.02
Fatigue	0.12***	0.03
Sleep	0.14***	0.02
Getting around	0.09***	0.02
Bathing/dressing	0.13***	0.03
Breathing	0.15***	0.02
Mouth sores	0.02	0.03
Eating	0.14***	0.02
Indigestion	0.09***	0.03
Constipation	0.10***	0.02
Diarrhoea	0.04	0.03

	Distress	
	**Coefficient**	**Std. Err.**
Changes in urination	0.07**	0.03
Fevers	-0.06	0.08
Dry/itchy skin	0.04	0.02
Dry/congested nose	0.03	0.03
Tingling in hands/feet	0.05**	0.02
Feeling swollen	0.08***	0.02
Sexual	0.03	0.02
**Other**		
Economic difficulties	0.11***	0.02
Legal problems	0.12***	0.03
Communication with health professionals	0.10***	0.03
Body image concerns	0.11***	0.02
Difficulties in obtaining information about the disease	0.09***	0.03

Note: **p<0.05; ***p<0.01

Table S2. Generalized linear model results for the relationship between patient characteristics and problem list, and distress.

	Distress	
	**Coefficient**	**Std. Err.**
**Demographics**		
Female	0.09***	0.03
**Disease phase**		
Treatment	Ref.	
Survival	0.00	0.03
Diagnosis	0.08	0.04
Relapse	0.06	0.04
Palliative	0.18***	0.06
**Practical**		
Housing	0.03	0.03
Security	0.01	0.03
Work/school	0.03	0.02
**Family**		
Dealing with partner	0.03	0.02
Dealing with children	0.05	0.02
**Emotional**		
Worry	0.11	0.07
Fears	0.02	0.04
Sadness	0.22***	0.05
Depression	0.16***	0.02
Nervousness	0.04	0.03
**Spiritual**		
Relating to God	0.01	0.03
Loss of Faith	0.04	0.03
**Physical**		
Pain	0.01	0.03
Nausea	0.02	0.02
Fatigue	-0.02	0.03
Sleep	0.08***	0.02
Getting around	-0.02	0.03
Bathing/dressing	0.05	0.03
Breathing	0.09***	0.03
Eating	0.05	0.03
Indigestion	-0.03	0.03
Constipation	0.01	0.02
Changes in urination	0.03	0.03
Tingling in hands/feet	-0.05	0.03
Feeling swollen	-0.04	0.02
Economic difficulties	0.01	0.03
Legal problems	0.05	0.04
Communication with health professionals	0.03	0.03
Body image concerns	0.01	0.02

	Distress	
	**Coefficient**	**Std. Err.**
Difficulties in obtaining information about the disease	0.00	0.03

Note: ***p<0.01

Table S3. Generalized linear model results for the relationship between patient characteristics and problem list (excluding emotional problems), and distress.

	Distress	
	**Coefficient**	**Std. Err.**
**Demographics**		
Female	0.10***	0.03
**Disease phase**		
Treatment	Ref.	
Survival	-0.01	0.03
Diagnosis	0.09**	0.04
Relapse	0.06	0.04
Palliative	0.14***	0.05
**Practical**		
Housing	0.02	0.03
Security	0.05	0.03
Work/school	0.05**	0.02
**Family**		
Dealing with partner	0.03	0.02
Dealing with children	0.06**	0.03
**Spiritual**		
Relating to God	0.01	0.04
Loss of Faith	0.07**	0.03
**Physical**		
Pain	0.03	0.03
Nausea	0.03	0.02
Fatigue	0.03	0.03
Sleep	0.10***	0.03
Getting around	-0.01	0.03
Bathing/dressing	0.06	0.03
Breathing	0.08***	0.03
Eating	0.07**	0.03
Indigestion	-0.02	0.03
Constipation	0.03	0.02
Changes in urination	0.03	0.03
Tingling in hands/feet	-0.05	0.03
Feeling swollen	-0.02	0.03
Economic difficulties		
Legal problems	0.03	0.03
Communication with health professionals	0.03	0.04
Body image concerns	0.04**	0.03
Difficulties in obtaining information about the disease	0.05	0.02

Note: **p<0.05; ***p<0.01
